# Tenapanor Beyond Phosphate: Gut Sodium Transport, Stool Transit Time, and Volume Balance in Dialysis Patients

**DOI:** 10.1111/hdi.70095

**Published:** 2026-05-31

**Authors:** Lucas Maciel de Almeida Corrêa, Luiggi Kevin Virgino Brandão, Clara Belo Gamon Santiago, Alexandre de Assis Barbosa, Gabriel Rian Mazur, Gabriel Costa de Santana, Yan Roberth Delmiro Silva, Letícia Esteves Dante, Ivan Felipe Dutra Júnior

**Affiliations:** ^1^ Faculdade de Medicina de São José do Rio Preto (FAMERP) São José do Rio Preto São Paulo Brazil; ^2^ Centro Universitário Uninorte (UNINORTE) Rio Branco Acre Brazil; ^3^ Universidade Estadual de Mato Grosso do Sul (UEMS) Campo Grande Mato Grosso do Sul Brazil; ^4^ Pontíficia Universidade Católica Do Paraná (PUCPR) Curitiba Paraná Brazil; ^5^ Universidade Salvador (UNIFACS) Salvador Bahia Brazil; ^6^ Universidade Federal de Alagoas (UFAL) Arapiraca Alagoas Brazil

**Keywords:** fibroblast growth factor‐23, hyperphosphatemia, renal dialysis, sodium–hydrogen exchanger 3 (NHE3), tenapanor, water–electrolyte balance

## Abstract

**Introduction:**

Tenapanor, a minimally absorbed intestinal NHE3 inhibitor approved for hyperphosphatemia in dialysis, lowers phosphate and reduces intestinal sodium absorption. Its sodium–water signal may have implications beyond phosphate but remains poorly separated from diarrhea‐mediated losses and dialysis prescription artifacts.

**Methods:**

This perspective synthesizes pharmacologic, trial, dialysis physiology, and CKD‐MBD evidence to propose a hypothesis‐generating framework for evaluating tenapanor beyond serum phosphate. We focused on intestinal sodium handling, stool transit, interdialytic weight gain, ultrafiltration stress, hemodynamic tolerance, and fibroblast growth factor 23.

**Findings:**

Tenapanor‐associated diarrhea and modest serum sodium reduction should be interpreted not only as tolerability events but also as potential markers of on‐target intestinal sodium–water modulation. In selected phenotypes, reduced effective sodium loading could theoretically attenuate thirst, lower interdialytic weight gain, and reduce ultrafiltration demand; in others, diarrhea‐mediated losses could worsen hypovolemia or intradialytic instability. Tenapanor‐induced fibroblast growth factor 23 reduction is biologically plausible but remains an unvalidated bridge to cardiovascular or patient‐centered outcomes.

**Discussion:**

Future studies should prospectively distinguish sodium–volume benefit from gastrointestinal intolerance using standardized dialysis endpoints, stool metrics, dialysate sodium data, bioimpedance, and imaging or patient‐centered outcomes. Tenapanor should therefore be studied as an intestinal sodium–phosphate–volume intervention, not merely a phosphate‐lowering drug.

**Abbreviations**
BID: twice a day (bis in die)
cFGF23: C‐terminal fibroblast growth factor 23
CKD: chronic kidney disease
CKD‐MBD: chronic kidney disease‐mineral and bone disorder
CV: cardiovascular
DOPPS: Dialysis Outcomes and Practice Patterns Study
FDA: US Food and Drug Administration
FGF23: fibroblast growth factor 23
FGFR4: fibroblast growth factor receptor 4
IDWG: interdialytic weight gain
iFGF23: intact fibroblast growth factor 23
KDIGO: Kidney Disease: Improving Global Outcomes
Na^+^
: sodium
NHE3: sodium–hydrogen exchanger isoform 3
RR: relative risk
UF: ultrafiltration

## 1 Introduction: The Phosphorus Paradox

Hyperphosphatemia in dialysis patients has been a therapeutic target for decades; yet, daily management remains disproportionately dependent on phosphate binders—a strategy that is biologically plausible but operationally fragile due to the high pill burden and the predictable erosion of adherence. Data from the Dialysis Outcomes and Practice Patterns Study (DOPPS) confirm that high pill burden correlates directly with non‐adherence and worse mineral metabolism outcomes [1]. This scenario helps explain the “phosphorus paradox”: despite successive generations of binders, the field has remained largely circumscribed to serum phosphorus as a surrogate endpoint, with little progress in intestinal mechanistic targets and patient‐centered metrics. Accordingly, this Perspective is hypothesis‐generating, proposing a pragmatic framework to disentangle on‐target sodium–volume modulation from diarrhea‐mediated losses and dialysis‐management artifacts.

This is where the plot twist emerges: tenapanor, an inhibitor of the sodium–hydrogen exchanger isoform 3 (NHE3) with predominantly luminal action, inaugurates a class with a distinct intestinal mechanism and a relatively predictable gastrointestinal profile, consistent with the increase of sodium and water in the stool [2, 3, 4]. The “timing statement” that justifies this Perspective is unequivocal: in the post‐approval era, the critical question is no longer just whether phosphorus decreases, but what else is being modulated—and how this modulation translates into tolerability, thirst, and hydrosaline balance in the dialysis patient [2].

While tenapanor is now approved for hyperphosphatemia in adults with CKD on dialysis in the United States (2023) and Japan (2023), and more recently in China (2025), access remains heterogeneous across health systems [2, 5, 6]. Nevertheless, the mechanistic lesson—coupling phosphate lowering to intestinal sodium handling via NHE3 inhibition—may inform a broader translational agenda that extends beyond a single product and toward next‐generation strategies targeting the sodium–phosphate–volume axis [4, 7].

## 2 The Sodium Signal: Noise or Physiology?

The current consensus is consistent: tenapanor reduces serum phosphorus in hemodialysis patients, including in combination strategies, at the cost of a higher frequency of gastrointestinal events—predominantly diarrhea—a profile aligned with its intestinal mechanism [4, 8, 9, 10, 11]. The one signal that deserves a more careful physiological reading is that a recent meta‐analysis, in addition to confirming a higher risk of diarrhea (RR ≈ 3.71), identified a statistically significant reduction in serum sodium (mean difference ≈ −0.7 mmol/L) [11].

If Na + falls—is this statistical noise or a relevant physiological signal? Although the magnitude is modest, in hemodialysis, small sodium shifts may reflect alterations in sodium/water balance, and therefore deserve to be decomposed before being discarded as an epiphenomenal finding [12, 13]. Importantly, a modest change in serum sodium does not preclude a clinically meaningful shift in total‐body sodium, given tight osmotic buffering and the dissociation between serum [Na^+^] and tissue sodium stores. In dialysis populations, ^23^Na‐MRI studies demonstrate dynamic sodium accumulation and removal from skin and muscle, supporting the concept that “exchangeable” and tissue sodium may change even when serum [Na^+^] moves little [13, 14, 15]. Three explanations compete: (i) real modulation of sodium balance by an on‐target intestinal effect (reduction of Na absorption, with systemic repercussions), (ii) a secondary effect mediated by diarrhea (hydrosaline losses and behavioral changes in intake), and (iii) artifacts of dialysis management (dialysate sodium prescription, ultrafiltration (UF), and pharmacological co‐interventions) [2, 11, 12]. The blind spot is methodological: available studies rarely collect, in a standardized and granular way, the variables necessary to discriminate these pathways—especially dialysate prescription data, interdialytic balance (weight gain, UF), thirst/intake metrics, and objective characterization of diarrhea [2, 8, 9, 10, 11, 12].

## 3 Reinterpreting Diarrhea: Adverse Event or Mechanism?

The field is under‐measuring what matters: tenapanor‐associated diarrhea has often been framed primarily as an “adverse event,” yet it is largely the clinical manifestation of the blockade of intestinal sodium absorption and the shift of water into the lumen [3, 4]. Clinically, however, it is essential to acknowledge that, for some patients, the unpredictability of bowel habits may be more burdensome than volume overload itself—so any putative sodium–volume benefit must be weighed against patient‐reported quality‐of‐life trade‐offs and tolerability [2, 8, 9, 10, 11]. In anuric patients, the dialysis machine concentrates volume and sodium removal, and the intestine becomes the main potential extracorporeal route for modulating hydrosaline balance in the interdialytic interval—an axis historically neglected in the discussion of hyperphosphatemia [12].

This same mechanism also reframes the discussion for patients who are constipated at baseline. Constipation is common in CKD and dialysis, driven by fluid restriction, reduced fiber intake, low physical activity, comorbid diabetes, and constipating medications, including phosphate binders; calcium carbonate and several other binders are particularly relevant because gastrointestinal intolerance and constipation may further worsen pill‐burden adherence [16, 17, 18]. Clinically, constipation is not a trivial symptom in dialysis: it has been associated with lower health‐related quality of life and, in observational hemodialysis cohorts, with higher all‐cause mortality and cardiovascular (CV) events [19, 20, 21]. Therefore, in a constipated phenotype, a tolerated increase in stool water content or bowel frequency could represent an adjunctive bowel‐function benefit rather than a side effect alone. Recent dialysis data support this possibility but remain hypothesis‐generating: one randomized trial reported improved stool consistency and reduced laxative use while maintaining phosphorus control, and a small prospective observational study suggested that tenapanor may allow reduction or discontinuation of stimulant laxatives in selected hemodialysis patients [22, 23]. Accordingly, tenapanor should not be presented as a laxative strategy in dialysis; the operational target is normalization of stool pattern while preserving phosphate control and avoiding clinically significant diarrhea.

A second undermeasured dimension is gut‐derived uremic solute biology. Slower intestinal transit and CKD‐related dysbiosis may favor the expansion of bacterial pathways that generate precursors of indoxyl sulfate and p‐cresyl sulfate, two protein‐bound uremic solutes that accumulate in dialysis and have been linked to adverse CV and renal phenotypes [18, 24]. Whether tenapanor‐induced changes in stool transit, luminal sodium‐water content, or bowel frequency alter these solutes has not been adequately tested in dialysis. This is precisely why future studies should measure indoxyl sulfate and p‐cresyl sulfate alongside stool frequency/consistency, laxative exposure, microbiome composition, phosphate control, and volume endpoints, rather than assuming that shorter transit automatically translates into lower gut‐derived toxin burden.

Although this Perspective focuses primarily on hemodialysis, the constipation signal is also relevant to peritoneal dialysis. Constipation is a recognized contributor to poor catheter drainage and catheter dysfunction, and management of bowel habit is part of the practical evaluation of a poorly draining peritoneal dialysis catheter [25]. Thus, if tenapanor is studied in peritoneal dialysis, bowel‐function outcomes should be interpreted not only as tolerability variables, but also as potential determinants of technique performance, drainage adequacy, and patient‐reported treatment burden.

In this context, tenapanor should be interpreted as an intervention in the phosphorus–sodium–water triad: by reducing intestinal Na^+^ absorption (with an increase in fecal Na^+^/water), it may decrease the effective systemic sodium load, attenuate the thirst drive, and consequently reduce interdialytic weight gain (IDWG)—which, in theory, would translate into a lower need for UF and greater hemodynamic tolerance to the session [3, 4, 12]. We acknowledge that thirst in hemodialysis is multifactorial (osmolar cues, neurohormonal activation, xerostomia, and learned behavior) and therefore may not track linearly with sodium balance; here, we posit that reducing effective interdialytic sodium loading is a physiologically plausible target to shift osmotic drive and thirst set‐point in selected phenotypes [12, 13]. This hypothesis, however, is bidirectional: if the effect is predominantly one of sodium balance, we would expect a reduction in thirst/IDWG and lower UF demand; if it is predominantly diarrhea‐driven, we would expect worse tolerability, risk of hypovolemia, and more intradialytic events [26, 27]. Thus, the “gastrointestinal signal” may represent hemodynamic benefit in some phenotypes and toxicity in others—and this physiological stratification has not yet been systematically incorporated into trials [2, 11]. This proposed intestinal sodium–volume axis and its potential systemic consequences are summarized in Figure 1.

**FIGURE 1:**
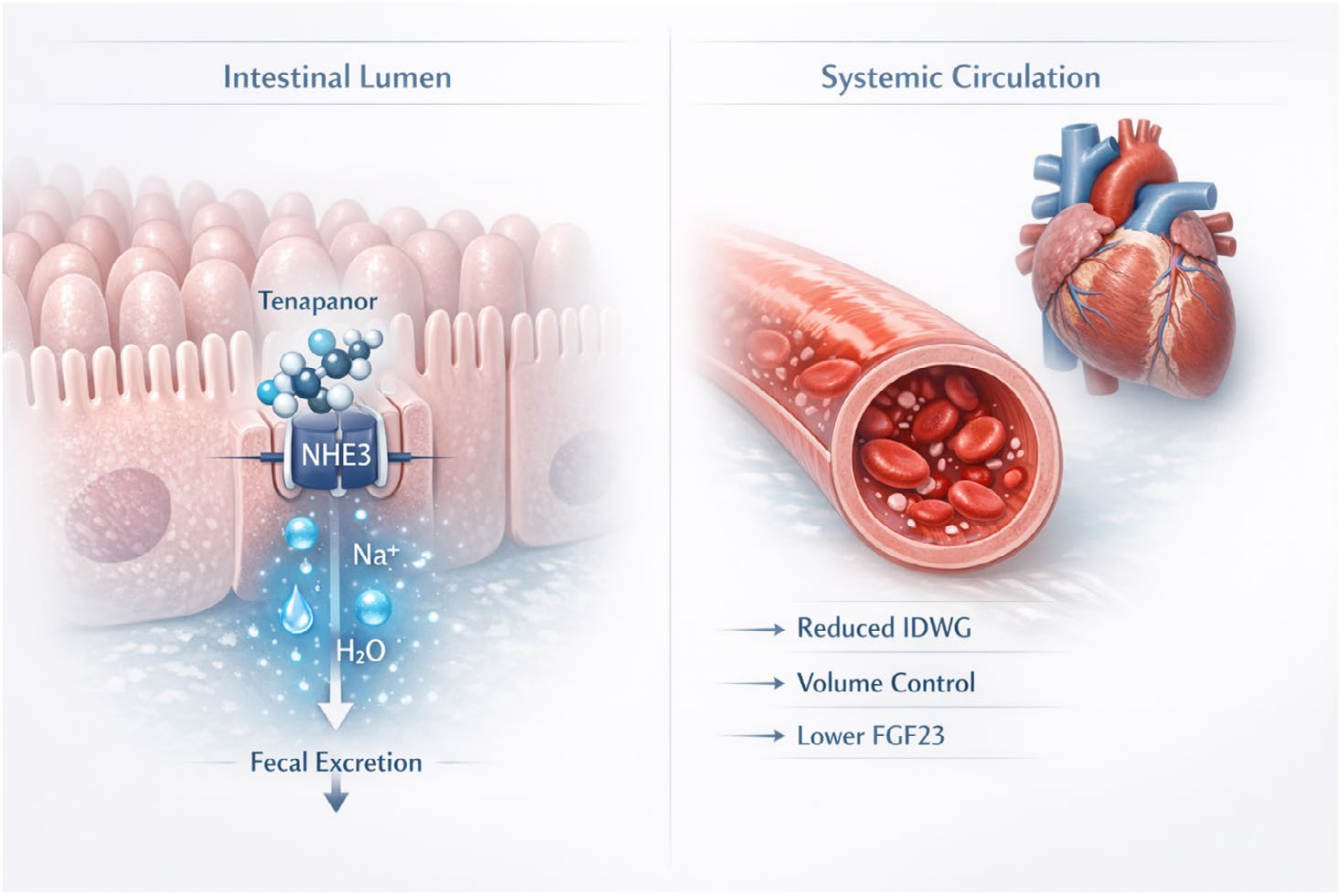
The tenapanor sodium–volume axis. Inhibition of intestinal NHE3 by tenapanor reduces sodium and water absorption, increasing fecal excretion. This mechanism is hypothesized to improve interdialytic volume control (reduced IDWG) and lower FGF23 levels, potentially mitigating CV stress beyond phosphate control.

## 4 Mechanism of Action and Clinical Translation

Tenapanor is a minimally absorbed inhibitor of the NHE3, expressed on the apical membrane of enterocytes. Its inhibition reduces transcellular sodium absorption, increases luminal retention of Na^+^ and water, and consequently promotes softer stools and higher bowel frequency—that is, a gastrointestinal phenotype that stems directly from the mechanism of action [3, 4]. Human pharmacodynamic studies consistently demonstrate this axis: increased fecal sodium excretion with concomitant reduction of urinary sodium excretion (in individuals with preserved renal function), in addition to an increase in stool weight/frequency [3]. In human epithelial models, NHE3 inhibition reduces the flux of Na^+^ and water and is coupled with a decrease in paracellular permeability to phosphate, providing a plausible mechanistic bridge for the serum phosphorus reduction observed in trials [7].

Thus, in hemodialysis, many gastrointestinal “adverse events” should be reinterpreted as on‐target effects—expected—of intestinal sodium/water modulation [2, 4]. However, being on‐target does not mean being clinically acceptable; it means that tolerability can (and must) be “engineered” by dose, titration, patient selection, and co‐intervention strategies [2]. From a practical prescribing standpoint, the approved regimen for adults with CKD on dialysis is tenapanor 30 mg orally twice daily, taken immediately before the morning and evening meals, with subsequent adjustment according to serum phosphorus response and gastrointestinal tolerability [2]. The label also advises that patients should not take tenapanor immediately before a hemodialysis session, but rather before the next meal after dialysis, reinforcing that dose timing is not a minor operational detail but a potential determinant of peak bowel effect and hemodynamic tolerability [2]. Timing remains mechanistically relevant: in phase 1, administration before meals increased fecal sodium excretion compared with administration after food, suggesting that synchronization with meals and dialysis sessions may modulate both therapeutic signal and adverse‐event profile [28].

## 5 The Missing Link: Volume and Hemodynamics

Although the mechanism of action points to a theoretical reduction in paracellular sodium absorption [4, 7], a critical gap persists in the literature: the absence of prospective studies designed specifically to evaluate standardized dialysis outcomes [8, 9, 10]. Critically, any net hemodynamic benefit would require that fecal sodium–water loss substitutes for (rather than adds to) interdialytic intake and is met by adaptive dialysis prescriptions (UF targets and dialysate sodium); otherwise, diarrhea‐mediated depletion could increase cramps, symptomatic hypovolemia, and intradialytic hypotension [26, 27]. While pivotal clinical trials, such as PHREEDOM and AMPLIFY, consolidated its efficacy in reducing serum phosphate, none of them used IDWG, UF rate, or bioimpedance parameters as robust primary or secondary outcomes [8, 9, 10]. This omission matters because intestinal sodium modulation is a double‐edged sword—potentially reducing thirst and volume overload, yet risking hemodynamic instability and intradialytic hypotension in patients with limited CV reserve—so without prospective, longitudinal assessment of interdialytic blood pressure and volume response, tenapanor's role beyond phosphorus control remains hypothesis rather than practice [12, 26, 27].

Although previous investigations have explored fecal sodium and IDWG, results did not demonstrate statistically significant differences over short observation periods [29]. However, this finding is far from closing the matter, since therapeutic windows of only four weeks, limited outcomes, and cohort heterogeneity obscure the drug's real potential [8, 10, 29]. It is imperative to recognize that the absence of a clinical signal may stem from uncontrolled methodological noise: behavioral adaptation, in which patients reduce fluid and dietary intake in response to induced diarrhea, and dialytic adaptation, where reactive adjustments in UF rate and dialysate conductivity mask the pharmacological effect on sodium balance [12, 27]. Therefore, progress in the clinical agenda does not depend on a proliferation of redundant trials focused purely on “phosphorus at 4 weeks,” but rather on robust, long‐duration study designs that capture real volume and hemodynamic dynamics under tenapanor use [12, 27, 29].

## 6 Safety by Design: Managing the Trade‐Off

Regulatory data for tenapanor show that the most frequent adverse event is diarrhea, generally mild to moderate, with rates varying between ~40% and 50% in clinical trials, while severe events, clinically relevant dehydration, and hyponatremia occurred with low frequency [2, 8, 9, 10]. These findings should not be dramatized but contextualized: the drug's mechanism implies, by definition, a potential impact on hydrosaline balance [3, 4]. In hemodialysis patients, whose volume homeostasis is already highly dependent on the interdialytic regimen and UF, any intervention that alters intestinal sodium dynamics requires a “safety by design” approach, structured by active monitoring and individualized titration [12, 26, 27]. We are not advocating for clinically significant diarrhea; rather, we propose titrating tenapanor toward a tolerated increase in fecal water content (i.e., mildly loosened stools rather than diarrheal illness) that may achieve net sodium offloading without symptomatic intolerance or hemodynamic compromise. This approach also hinges on patient education and shared decision‐making, so patients can distinguish expected, mildly loosened stools from pathologic diarrhea and promptly report “red‐flag” symptoms. Because phosphate lowering is dose‐dependent in the clinical program, “engineered tolerability” should be operationalized as titration that preserves phosphorus targets rather than compromises them [8, 9, 10]. When gastrointestinal tolerability limits monotherapy, a pragmatic strategy is combination with lower‐dose binders to maintain phosphate control while minimizing pill burden and avoiding clinically significant diarrheal illness [10].

In practice, four operational components can make use more predictable. (1) Baseline: assess serum sodium, interdialytic blood pressure, history of intradialytic hypotension, clinical frailty, prior gastrointestinal pattern, especially baseline constipation, irritable bowel syndrome, stool frequency/consistency, and concomitant laxative use [2, 8, 16, 17, 22, 23, 26, 27, 29]. (2) Initial phase (2–4 weeks): monitor sodium and clinical signs of volume, especially in patients with greater interdialytic weight variation. (3) Red flags: persistent diarrhea, dizziness, sustained drop in interdialytic systolic pressure, or progressive downward trend in serum sodium. (4) Stop rules: temporary pause or discontinuation in the face of confirmed hyponatremia, clinically relevant hypovolemia symptoms, or persistent gastrointestinal intolerance, with cautious reintroduction only after clinical re‐equilibration. In this framework, risk is not denied; it is systematically anticipated and managed.

## 7 FGF23: The Bridge Between Biology and Outcomes

If the therapeutic goal in chronic kidney disease‐mineral and bone disorder (CKD‐MBD) is to reduce systemic toxicity, focusing exclusively on the phosphorus number is insufficient. Fibroblast growth factor 23 (FGF23) has emerged as a plausible integrating node between phosphate overload, CV remodeling, and mortality in dialysis, with elevated levels consistently associated with left ventricular hypertrophy, cardiac dysfunction, and higher risk of events and death [30, 31, 32]. Phosphorus reductions with different interventions are frequently accompanied by FGF23 reduction, including with tenapanor, suggesting a possible biological impact beyond isolated biochemical control [8, 10].

Importantly, the field has learned—often painfully—that biomarker plausibility does not guarantee clinical translation. Even when interventions improve CKD‐MBD biology, downstream CV benefit may be inconsistent or diluted by competing risks and co‐interventions. For example, cinacalcet has been associated with reductions in FGF23 in ESRD, yet the EVOLVE trial did not demonstrate a clear, uniform reduction in hard CV outcomes in the primary analysis—highlighting how “biochemical wins” can fail to map neatly onto patient‐level events. Likewise, phosphate‐lowering strategies in non‐dialysis CKD have not reliably translated into improved clinical outcomes. Accordingly, tenapanor's FGF23 reduction should be framed as a testable bridge hypothesis—requiring prospective validation against imaging‐based surrogates and patient‐centered endpoints rather than being treated as an intrinsic value marker [33, 34, 35]. Even if CV translation proves neutral, characterizing the FGF23 response may still be clinically informative within the broader CKD‐MBD milieu (as a mechanistic readout and potential mediator of extra‐cardiac phenotypes), reinforcing the need to anchor this biomarker signal to adjudicated clinical and imaging‐based endpoints rather than presume benefit.

A rare and potentially relevant biological signal emerged from the tenapanor clinical program in hemodialysis: in addition to the drop in phosphorus, there was a significant reduction in FGF23 within a few weeks compared to placebo [8, 10]. This observation is attractive because FGF23 has mechanistic plausibility for participating in CV damage, including pro‐hypertrophic myocardial effects described in experimental models [30]. Crucially, FGF23 levels are also influenced by volume status and sodium load, creating a potential synergistic benefit where tenapanor could lower FGF23 through both phosphate‐dependent and volume‐dependent pathways [12, 36].

The problem is that the “so what?” remains unanswered: we do not know if reducing FGF23 with tenapanor alters vascular calcification, arterial stiffness, left ventricular hypertrophy trajectory, or bone outcomes, nor if this signal remains consistent when inserted into real‐world care, where calcimimetics and vitamin D modulate the CKD‐MBD axis and may shift FGF23 in different directions [33, 37]. Instead of treating FGF23 as a final endpoint, the next step should test the hypothesis operationally: (1) a pragmatic trial embedded in a registry, with randomization integrated into the routine of large dialysis networks and passive event capture, capable of scaling sample size and representativeness without prohibitive costs; and (2) a patient‐centered composite endpoint, combining hospitalization, CV events, and fracture, with feasible follow‐up (12–24 months) and mechanistic substudies (vascular imaging and echocardiography) to connect the “biochemical signal” to what truly matters to the patient.

## 8 A Proposal for the Future Agenda

The CKD‐MBD field in dialysis already has clarity that controlling phosphorus is necessary, but it is still insufficient to say that an intervention “lowered phosphorus” if it also affects the sodium–volume axis and may, through this path, alter thirst, IDWG, UF stress, and intradialytic hypotension [12, 26, 27, 37]. Tenapanor, by being an intestinal NHE3 inhibitor with increased fecal sodium/water content and diarrhea as the main adverse event, offers a “sodium signal,” a predictable, on‐target shift in intestinal sodium–water handling—whose post‐approval question is whether modest sodium offloading translates into lower IDWG/UF stress rather than diarrhea‐mediated losses and hemodynamic instability [2, 3, 4, 8, 11, 29].

Thus, we propose two feasible studies as a blueprint:

1. Pragmatic trial (≥ 3–6 months) with dialysis outcomes and safety—Population: hemodialysis patients with high IDWG and/or refractory phosphorus despite binder escalation, enriching with a phenotype of intradialytic hypotension history; Intervention: tenapanor with a practical strategy (e.g., 30 mg BID with titration guided by intestinal tolerability and adherence), incorporating standard diet and dialysis management; Comparator: “escalate phosphate binders”/usual care; Primary endpoint: change in IDWG (kg or % of dry weight) and/or UF stress (UF rate mL/kg/h), with two secondaries: bioimpedance (hyper/hypovolemia, extracellular water) and incidence of intradialytic hypotension/hyponatremia and discontinuation due to adverse events [38, 39]. “Make‐or‐break” subgroups: low versus normal baseline Na; high versus low dialysate sodium and/or history of intradialytic hypotension.
2. Mechanistic study linking FGF23 to imaging/vascular stiffness—Population: hemodialysis with persistent hyperphosphatemia and elevated FGF23; Intervention: tenapanor (isolated or as add‐on, per local practice); Comparator: intensification of binders/usual care; Primary endpoint: variation in FGF23 (iFGF23 and/or cFGF23) and its association with vascular stiffness measures by imaging (e.g., pulse wave velocity, aortic distensibility) and cardiac remodeling; two secondaries: left ventricular mass and hemodynamic/volume markers. Rationale: FGF23 is not just a “number,” as there is mechanistic and translational evidence that it may promote ventricular hypertrophy via FGFR4 signaling, which makes the “biomarker‐CV phenotype” bridge a testable and clinically relevant hypothesis [30, 40].

In the end, the question that must close the agenda is an explicit trade‐off: if tenapanor improves volume and UF but worsens intradialytic hypotension and discontinuation, which exchange do we accept—and for whom? Taken together, these considerations are summarized in Table 1, which organizes the established or observed signals, unresolved uncertainties, and practical implications of tenapanor beyond phosphate control across dosing, gastrointestinal tolerability, constipation, gut‐derived uremic solutes, peritoneal dialysis relevance, sodium–volume endpoints, and the FGF23 bridge hypothesis.

**TABLE 1:** Summary of tenapanor signals beyond phosphate control in dialysis.

Domain	Established or observed signal	Key uncertainty	Practical implication
Phosphate control and pill burden	Phosphate‐binder pill burden is associated with nonadherence and worse mineral metabolism; tenapanor lowers serum phosphate in dialysis, including as add‐on to binders [1, 2, 8, 9, 10, 11].	Whether phosphate lowering and any potential reduction in binder burden translate into patient‐centered CKD‐MBD benefit independent of other co‐interventions remains unproven [33, 34, 35, 37].	Use tenapanor as a phosphate‐lowering therapy; treat benefits beyond phosphate as hypotheses requiring outcome validation.
Dose and administration	Approved adult regimen is 30 mg orally twice daily immediately before morning and evening meals; dose timing relative to meals changes fecal sodium pharmacodynamics, and dosing immediately before HD should be avoided [2, 28].	Optimal synchronization with meals, dialysis sessions, and titration to preserve efficacy while limiting bowel or hemodynamic intolerance is unknown.	Report dose, titration, meal timing, and timing relative to HD in future studies and clinical descriptions.
NHE3 mechanism and fecal sodium‐water axis	Tenapanor is minimally absorbed; intestinal NHE3 inhibition increases luminal sodium‐water retention, stool weight/frequency, and reduces paracellular phosphate permeability [3, 4, 7].	The net clinical signal may represent sodium offloading, diarrhea‐mediated losses, dialysis‐management artifacts, or all three.	Measure stool pattern, serum sodium, dialysate sodium, IDWG, UF rate, and BP together rather than interpreting diarrhea in isolation.
Gastrointestinal tolerability	Diarrhea is the dominant adverse event in trials and meta‐analysis, usually mild to moderate but relevant for discontinuation and adherence [2, 8, 9, 10, 11].	Prospective phenotypes separating useful stool softening from intolerance have not been defined.	Use engineered tolerability: education, early monitoring, individualized titration, and stop rules for persistent diarrhea, hypovolemia, or worsening hyponatremia.
Constipation phenotype	Constipation is common in CKD/dialysis, worsens HRQoL, and is linked with adverse outcomes; recent dialysis studies suggest improved stool consistency and reduced laxative exposure with tenapanor [16, 17, 18, 19, 20, 21, 22, 23].	Sustained effects on quality of life, hospitalization, cardiovascular events, or bowel‐related complications remain unproven.	Do not present tenapanor as a laxative; target stool‐pattern normalization while maintaining phosphate control and avoiding clinically significant diarrhea.
Gut‐derived uremic solutes	CKD‐related dysbiosis and slower transit may favor indoxyl sulfate and p‐cresyl sulfate generation; these protein‐bound solutes accumulate in dialysis [18, 24].	Whether tenapanor changes these solutes through transit, luminal sodium‐water content, or microbiome shifts has not been adequately tested.	Measure IS, PCS, microbiome markers, stool pattern, laxative exposure, phosphate, and volume endpoints together.
Peritoneal dialysis relevance	Constipation contributes to poor PD catheter drainage and catheter dysfunction [25].	Tenapanor‐specific effects on PD drainage, catheter performance, technique burden, and technique survival remain unstudied.	PD studies should prespecify bowel habit, drainage adequacy, catheter function, and patient‐reported treatment burden.
Sodium‐volume and hemodynamic axis	Sodium balance is central in dialysis, tissue sodium may change despite small serum sodium shifts, and IDWG, UF rate, bioimpedance, and BP are clinically meaningful dialysis outcomes; short‐term tenapanor data have not shown a clear IDWG signal [12, 13, 14, 15, 26, 27, 29, 38, 39].	Whether tenapanor reduces thirst, IDWG, and UF stress or increases hypovolemia, intradialytic hypotension, or hyponatremia is unresolved [2, 11, 12, 26, 27, 29].	Test the sodium‐volume hypothesis using IDWG, UF rate, bioimpedance, interdialytic BP, intradialytic hypotension, serum sodium, and discontinuation as core endpoints.
FGF23 and CKD‐MBD bridge hypothesis	Tenapanor can reduce FGF23; FGF23 is linked to LVH and mortality, but prior CKD‐MBD biomarker improvements have not reliably translated into hard cardiovascular benefit [8, 10, 30, 31, 32, 33, 34, 35, 37, 40].	Whether tenapanor‐related FGF23 reduction improves vascular stiffness, LVH trajectory, fracture risk, hospitalization, or cardiovascular outcomes is unknown.	Frame FGF23 as a mechanistic bridge endpoint, not a surrogate victory; pair biochemical endpoints with imaging‐based and patient‐centered outcomes.

Abbreviations: BID, twice daily; BP, blood pressure; CKD, chronic kidney disease; CKD‐MBD, chronic kidney disease‐mineral and bone disorder; FGF23, fibroblast growth factor 23; HD, hemodialysis; HRQoL, health‐related quality of life; IDWG, interdialytic weight gain; IS, indoxyl sulfate; LVH, left ventricular hypertrophy; NHE3, sodium‐hydrogen exchanger isoform 3; PCS, p‐cresyl sulfate; PD, peritoneal dialysis; UF, ultrafiltration.

## 9 Conclusion

In summary, tenapanor has already demonstrated phosphorus reduction (and, in longer studies, sustained it), including in combined strategies with binders, but the field has not yet adequately tested whether the “sodium signal” is a risk, a benefit, or both—depending on the phenotype [8, 9, 10]. Likewise, observed reductions in FGF23 need to stop being just “elegant biology” and become a causal bridge hypothesis for measurable CV outcomes by imaging, especially if we intend to justify value beyond the laboratory [30, 40].

If it demonstrates sustained reduction of IDWG/UF stress without increased intradialytic hypotension or hyponatremia, tenapanor can be repositioned as a volume management tool beyond phosphorus. If the mineral benefit comes at the cost of hemodynamic instability and discontinuation, clinical value will be restricted to carefully selected phenotypes. The call to action, therefore, is to move tenapanor from a “phosphorus reducer” to an intervention of patient‐centered value, anchored in safety, tolerability, and sustained dialysis outcomes [2, 8, 9, 10, 11, 12, 26, 27, 29].

## Funding

The authors have nothing to report.

## Ethics Statement

The authors have nothing to report.

## Conflicts of Interest

The authors declare no conflicts of interest.

## Data Availability

Data sharing not applicable to this article as no datasets were generated or analysed during the current study.
